# Alternative splicing of FBP-interacting repressor coordinates c-Myc, P27Kip1/cyclinE and Ku86/XRCC5 expression as a molecular sensor for bleomycin-induced DNA damage pathway

**DOI:** 10.18632/oncotarget.1650

**Published:** 2013-12-21

**Authors:** Bahityar Rahmutulla, Kazuyuki Matsushita, Mamoru Satoh, Masanori Seimiya, Sachio Tsuchida, Shuji Kubo, Hideaki Shimada, Masayuki Ohtsuka, Masaru Miyazaki, Fumio Nomura

**Affiliations:** ^1^ Department of Molecular Diagnosis, Graduate School of Medicine, Chiba University, Chiba City, Chiba, Japan; ^2^ Division of Laboratory Medicine, Chiba University Hospital, Chiba City, Chiba, Japan; ^3^ Department of General Surgery, Graduate School of Medicine, Chiba University, Chiba City, Chiba, Japan; ^4^ Department of Orthopedics and Traumatology, Hospital of Uyghur Medicine, Urumqi, Sinkiang Uyghur Autonomous Region, P. R. China; ^5^ Department of Genetics, Hyogo College of Medicine, Nishinomiya City, Nishinomiya, Hyogo Prefecture, Japan; ^6^ Department of Gastroenterological Surgery, Toho University Omori Medical Center, Ota-ku, Tokyo, Japan

**Keywords:** FBP interacting repressor (FIR), bleomycin(BLM), DNA damage, Ku86, c-Myc, P27Kip1

## Abstract

The far-upstream element-binding protein-interacting repressor (FIR) is a *c-myc* transcriptional suppressor. FIR is alternatively spliced to lack the transcriptional repression domain within exon 2 (FIRΔexon2) in colorectal cancers. FIR and FIRΔexon2 form homo- or heterodimers that complex with SAP155. SAP155, a subunit of the essential splicing factor 3b subcomplex in the spliceosome, is required for proper P27Kip1 pre-mRNA splicing, and P27Kip1 arrests cells at G1. In contrast, FIR was co-immunoprecipitated with Ku86 and DNA-PKcs. siRNA against Ku86/Ku70 decreased FIR and P27Kip1 expression, whereas siRNA against FIR decreased Ku86/XRCC5 and P27Kip1 expression. Thus the mechanical interaction of FIR/FIRΔexon2/SAP155 bridges *c-myc* and P27Kip1 expression, potentially integrates cell-cycle progression and *c-myc* transcription in cell.

Bleomycin (BLM) is an anticancer agent that introduces DNA breaks. Because DNA breaks generate the recruitment of Ku86/Ku70 to bind to the broken DNA ends, the possible involvement of FIR and Ku86/Ku70 interaction in the BLM-induced DNA damage repair response was investigated in this study. First, BLM treatment reduced SAP155 expression and increased FIR and FIRΔexon2 mRNA expression as well as the ratio of FIRΔexon2:FIR in hepatoblastoma cells (HLE and HLF). Second, FIR or FIRΔexon2 adenovirus vectors (Ad-FIR or Ad-FIRΔexon2) increased Ku86/Ku70 and P27Kip1 expression *in vitro*. Third, BLM decreased P27Kip1 protein expression, whereas increased P27Kip1 and γH2AX expression with Ad-FIRΔexon2. Together, the interaction of FIR/SAP155 modulates FIR splicing and involves in cell-cycle control or cell fate via P27Kip1 and *c-myc* in BLM-induced DNA damage pathway. This novel function of FIR splicing will contribute to clinical studies of cancer management through elucidating the mechanical interaction of FIR/FIRΔexon2/SAP155 as a potential target for cancer treatment.

## INTRODUCTION

The far-upstream element (FUSE) is a sequence required for proper transcriptional regulation of the human *c-myc* gene [1,2]. FUSE is located 1.5-kb upstream of the *c-myc* promoter P1 and is recognized by the FUSE-binding protein (FBP). FBP is a transcription factor that stimulates *c-myc* expression through FUSE [2,3]. FBP and the FUSE-binding protein-interacting repressor (FIR) have been reported to be a sensor of DNA melting of *c-myc* promoter, and regulate *c-myc* transcription through the general transcription factor TFIIH [2,4-8]. Yeast two-hybrid analysis has demonstrated that FBP binds to FIR, and FIR represses *c-myc* transcription by suppressing the TFIIH/P89/XPB helicase (P89)[4,8]. Cells from Type B and Type D xeroderma pigmentosum patients are defective in FIR repression, which suggests that P89 mutations impair *c-myc* transcriptional regulation by FIR and contribute to tumor development [5]. Expression of FIRΔexon2, an FIR splice variant that lacks exon 2, may promote tumor development by disabling FIR repression of *c-myc* [9].

Splicing factor 3b (SF3b) is a subcomplex of the U2 small nuclear ribonucleoprotein in the spliceosome [10]. SAP155 (subunit of SF3b) is required for proper FIR expression and vice versa, and SAP155 knockdown or SF3b inhibition disrupts alternative splicing of FIR pre-mRNA and generates FIRΔexon2 [11]. Therefore, a complex formation of SAP155 with FIR/FIRΔexon2 disturbs well-established functions of SAP155 and FIR, serving as a molecular switch for *c-myc* gene expression [11]. In cancers, cell-cycle arrest for complete DNA damage repair is highly inefficient because expression of the Cip/Kip family is decreased; thus, cell-cycle progression is accelerated [12,13]. Together, interaction between FIR/FIRΔexon2 and SAP155 bridges *c-myc*/*c-Myc* expression and cell cycling. Because FIR/FIRΔexon2/SAP155 interaction connects *c-myc* and cell-cycle regulation by integrating the expression of P89/FIR/FIRΔexon2 or P27/cdk2/cyclinE [14], FIR potentially plays some role in DNA-damage responses [14,15]. Bleomycin (BLM) produces much higher levels of DNA double strand breaks (DSBs) with relatively uniform and simple DNA ends [16,17]. Single-strand DNA breaks (SSDs) lead to DSBs that occur in close proximity and are produced with higher concentrations of BLM [18-20]. DSBs are one of the most severe types of DNA damage and they promote genomic instability that is lethal to the cell if left unrepaired [21,22]. Several different DNA repair pathways combat DSBs, with nonhomologous end joining (NHEJ) being one of the major pathways in mammalian cells [21,23]. The core components of mammalian NHEJ are the catalytic subunit of DNA protein kinase (DNA-PKcs), Ku70/Ku80, Artemis, XRCC4, and DNA ligase IV [21]. End bridging occurs via interactions between the DNA-PKcs molecules, leading to DSB repair [24]. The purpose of this study was to reveal FIR's novel potential role in DNA damage repair pathway by studying how FIR coordinates, integrates or orchestrates BLM-induced DNA-damage responses. The results we obtained indicated that FIR and Ku86/Ku70 potentially form complexes and participate in BLM-induced DNA-damage repair machinery. The possible interactions of FIR/FIRΔexon2/SAP155 and Ku86/Ku70/DNA-PKcs may provide new insight into DNA damage response pathway of cells. The importance of the FIR/FIRΔexon2/SAP155 interaction is discussed as a novel modulator of *c-myc*/cell-cycle checkpoint regulator in cancer development or future clinical applications [25].

## RESULTS

### FIR/FIRΔexon2/SAP155, Ku86/XRCC5, and DNA-PKcs potentially form a complex

**Figure 1:**
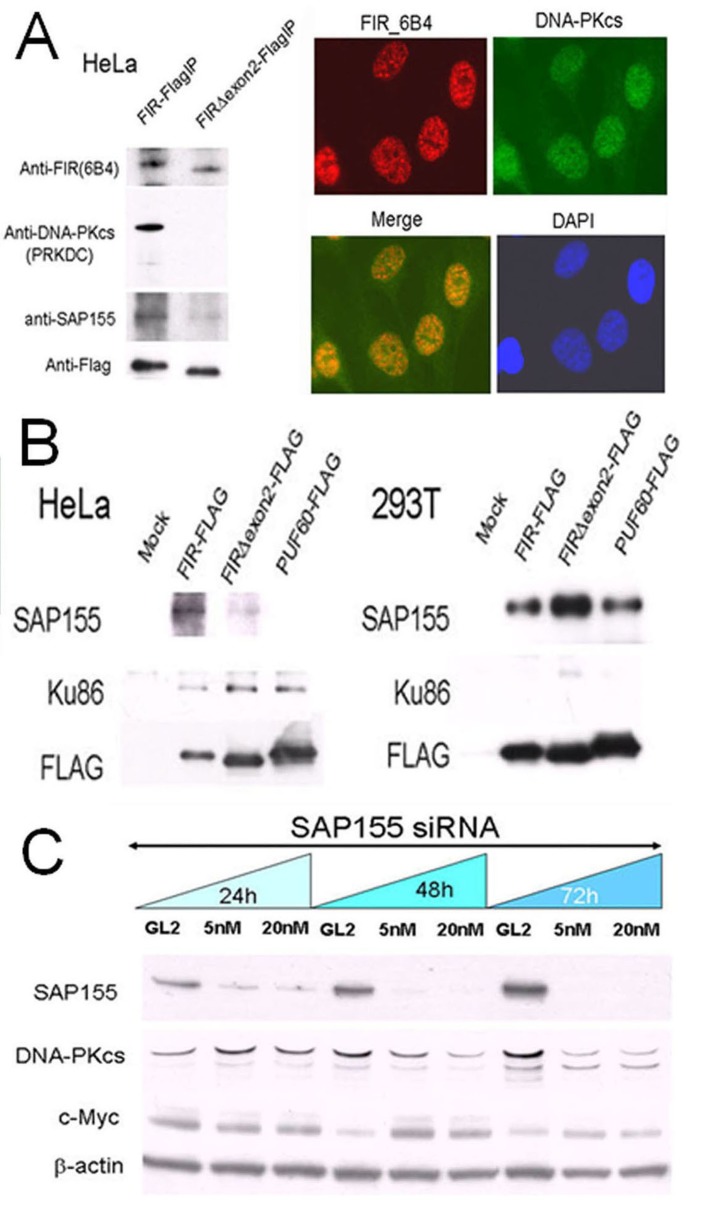
FIR/SAP155 and Ku86/DNA-PKcs potentially form a complex *in vitro* (A) DNA-PKcs and SAP155 were examined by pull-down assays in HeLa cells stably expressing FIR–FLAG or FIRΔexon2–FLAG. Typical immunohistochemical stainings with FIR and DNA-PKcs antibodies are shown. (B) Pull-down assays were performed for Ku86 and SAP155 in HeLa and 293T cells transiently expressing FIR–FLAG, FIRΔexon2–FLAG, or PUF60–FLAG. Mock is the negative control with the empty vector. (C) HeLa cells were treated for 24, 48, and 72 h with 5-nM or 20-nM siRNA against SAP155. SAP155, DNA-PKcs, and c-Myc were examined by Western blotting. The negative control was siRNA against GL2.

If FIR plays some role in the DNA-damage response, FIR potentially forms a complex with DNA-damage repair proteins such as DNA-PKcs or Ku86/Ku70. Under this hypothesis, we prepared FIR–FLAG, FIRΔexon2–FLAG or PUF60–FLAG stably expressing HeLa cells (cancer cells) or 293T cells (non-cancer cells), and pull-down assays were performed to examine whether FIR, DNA-PKcs and Ku86 interacted. Consequently, DNA-PKcs was co-immunoprecipitated in stably expressing FIR–FLAG, but not in those expressing FIRΔexon2–FLAG (Figure 1A). Ku86 has been reported to form a complex with DNA-PKcs/PARP1 to participate in NHEJ of DNA DSB repair [26]. Indeed, Ku86 was co-immunoprecipitated with FIR from HeLa cells but not from 293T cells (Figure 1B). Therefore, pull-down assays indicated that FIR possibly interacts with DNA-PKcs and Ku86 in Hela cells (Supplementary Table 1; Figure 1C). Because SAP155 was co-immunoprecipitated with FIR (Figure 1B) and SAP155 affects proper FIR pre-mRNA splicing [11], the interaction between SAP155 and DNA-PKcs was further investigated. SiRNA against SAP155 significantly suppressed DNA-PKcs and activated *c-myc* (Figure 1C). Together, these results indicate that FIR/SAP155 potentially interacts with Ku86/Ku70/DNA-PKcs or forms complex at least in HeLa cells.

### Total FIRs, SAP155 and Ku86 were upregulated in human hepatocellular carcinoma (HCC) tissues

**Figure 2:**
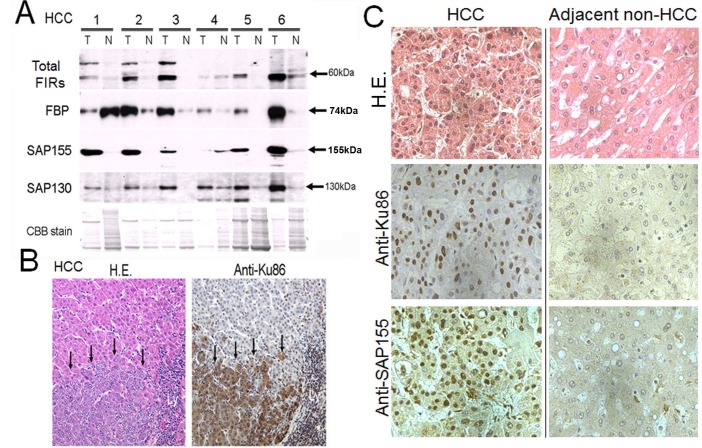
FIR, SAP155, and Ku86 were upregulated in human hepatocellular carcinoma (HCC) tissues (A) Expression of FIR and related proteins was examined by Western blotting in six paired tumor (T) and adjacent non-tumor (N) tissue samples from HCC tissues. A typical IHC staining of HCC tissues against anti-Ku86 antibody in lower magnification (B) and high power fields of anti-Ku86 and anti-SAP155 (C) were shown.

Given FIR/SAP155 forms a complex with Ku86, their expression levels should be coordinately regulated in the same direction. Expectedly, FIR, SAP155 (Figure 2A), and Ku86 (Figure 2B) were significantly upregulated in excised human HCC tissues compared with adjacent non-cancer tissues (Figure 2C). Notably, Ku86 was upregulated at protein level in HCC [27]. FIR has been reported to be engaged in cell-cycle regulation through P27Kip1 expression by affecting SAP155 function [14]. These results indicate that FIR, SAP155, and Ku86 also form a complex *in vivo* (in tissues) and disturb DNA-damage repair in HCC. Therefore, we examined whether altered FIR expression potentially influenced the expression levels of DNA repair proteins and BLM-induced DNA-damage repair.

### Altered FIR/FIRΔexon2 expression changes DNA-damage repair protein Ku86 and vice versa

**Figure 3:**
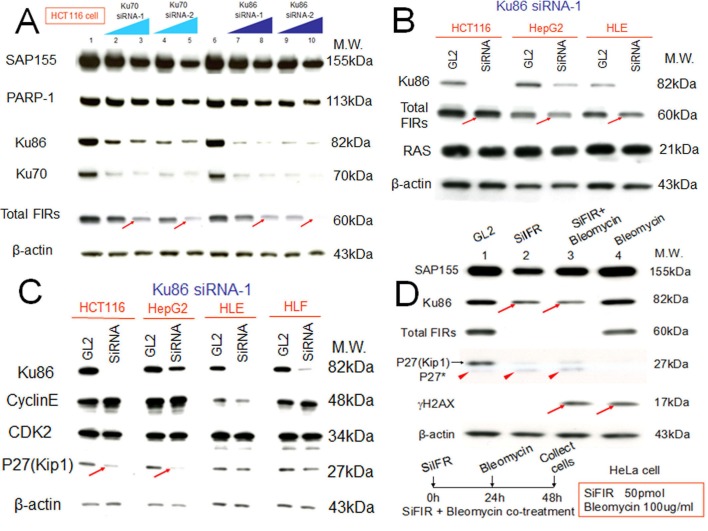
Knockdown of Ku86 with siRNA decreased FIR and vice versa FIR and Ku86 expression was examined after treatment of HCT116, HepG2, and HLE cell lines with the respective siRNAs. GL2 siRNA was transfected as the negative control. After 48 h of incubation, whole-cell extracts were analyzed by Western blotting. (A) Two types of Ku86 and Ku70 siRNAs were transfected into HCT116 cells. Lanes 1 and 6 are GL2 siRNA control transfections, lanes 2–5 are Ku70 siRNA transfections, and lanes 7–10 are Ku86 siRNA transfections. Lanes 2 and 7 were with 40 pmol of type 1 siRNA (Ku70 siRNA-1 or Ku86 siRNA-1), lanes 3 and 8 were with 80 pmol of type 1 siRNA (Ku70 siRNA-1 or Ku86 siRNA-1), lanes 4 and 9 were with 40 pmol of type 2 siRNA (Ku70 siRNA-2 or Ku86 siRNA-2), and lanes 5 and 10 were with 80 pmol of type 2 siRNA (Ku70 siRNA-2 or Ku86 siRNA-2). (B) HCT116, HepG2, and HLE cells were transfected with 50 pmol of type 1 Ku86 siRNA (Ku86 siRNA-1). (C) Ku86 type 1 siRNA (Ku86 siRNA-1) was transfected into four cell lines at different concentrations: 50 pmol for HCT116 and HepG2 cells and 10 pmol for HLE and HLF cells. (D) HeLa cells were treated with 50 pmol of FIR siRNA (SiFIR) and/or 100 μg/ml BLM. BLM was added to cells 24 h after FIR siRNA transfection. After a total of 48 h of incubation, cell lysates were extracted and analyzed for protein expression of Ku86, FIR, P27Kip1, and γH2AX. Lane1 is the GL2 siRNA control. P27*, a splicing variant of P27, is indicated by arrowheads.

**Figure 4:**
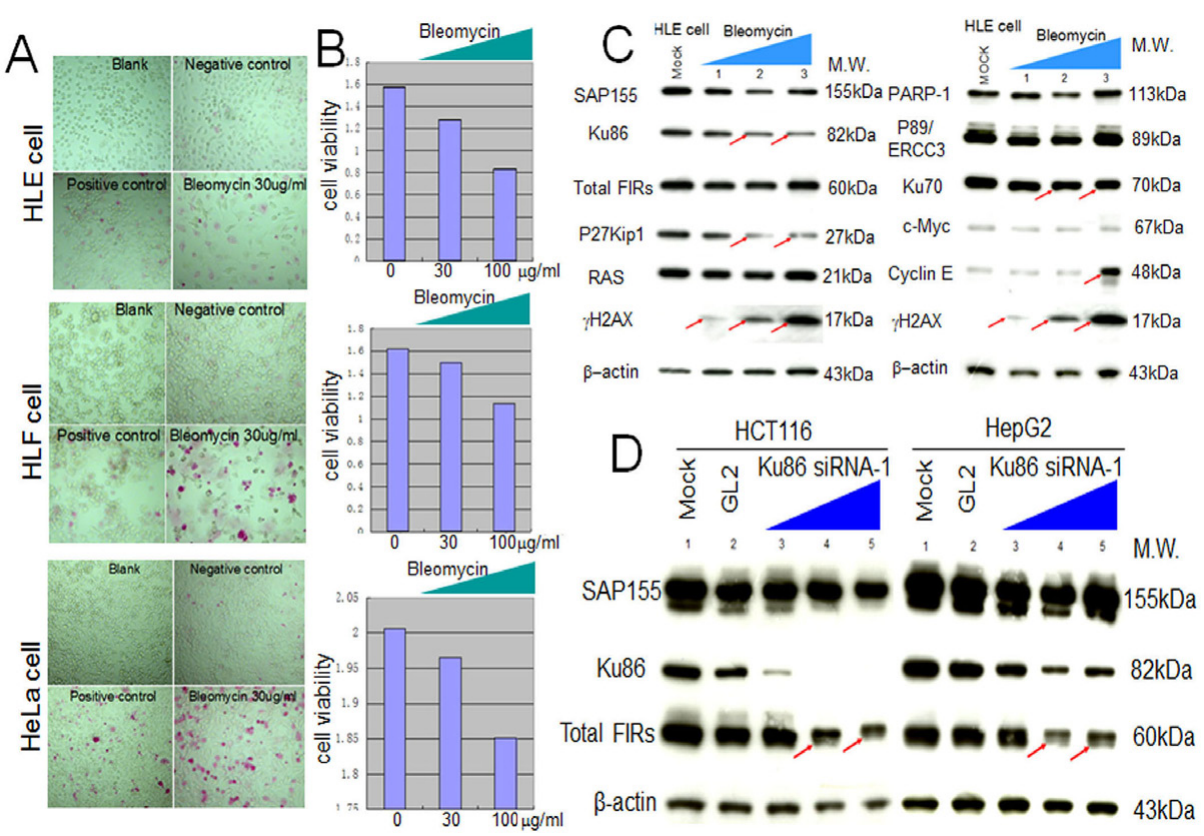
Apoptotic effect of BLM in HLE, HLF, and HeLa cells To examine the apoptotic effect of BLM, apoptosis (A) and MTS assays (B) were performed. (C) HLE cells were treated with BLM and subjected to western blotting (lanes1–3: 10, 100, 200 μg/ml for 48 hr, respectively). Mock is untreated cell lysate as negative control. (D) Type1 Ku86 siRNA was transfected into HCT116 and HepG2 cells, and western blotting was performed; lanes 1, untreated cell lysates; lanes 2, GL2 siRNA control transfections; lanes 3–5, 10, 50, and 100 pmol of Ku86 siRNA, respectively.

**Figure 5:**
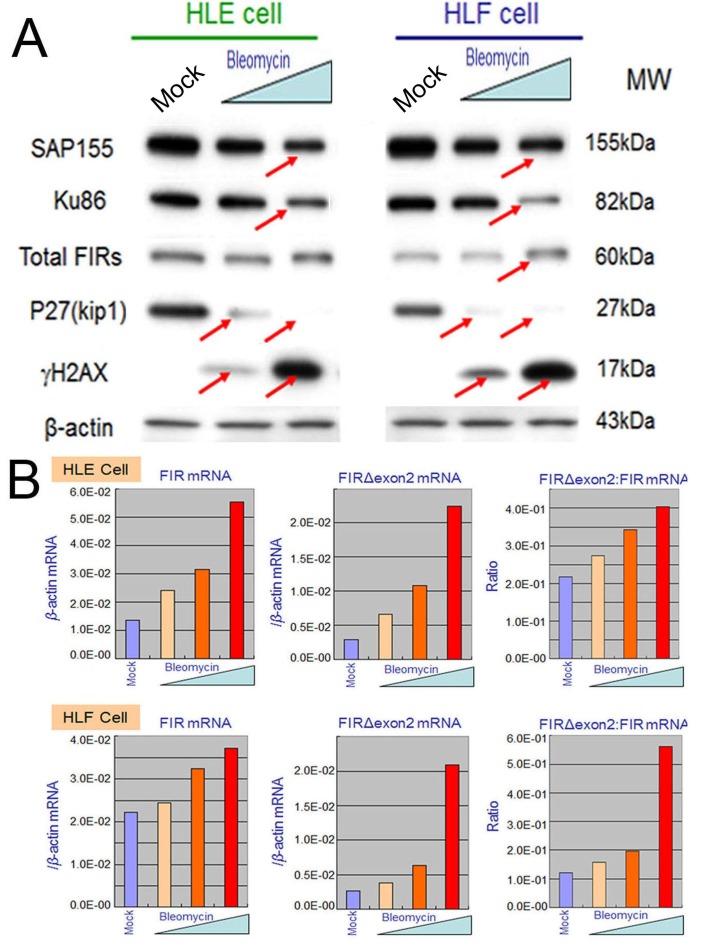
BLM treatment decreased SAP155 and significantly increased FIR and FIRΔexon2 mRNA expression as well as the FIRΔexon2:FIR ratio in hepatoblastoma (HLE and HLF) cells (A) HLE and HLF cells were treated for 48 h with 10 and 100 μg/ml of BLM. whole-cell extracts were analyzed by Western blotting. Mock is untreated cells as negative control. (B) HLE and HLF cells were treated for 48 h with 1, 10, 100 μg/ml of BLM. Total RNAs extracted from the cells were converted to cDNAs and quantitative RT-PCR was performed for FIR and FIRΔexon2 mRNA expression. Mock is untreated cells as negative control.

**Figure 6:**
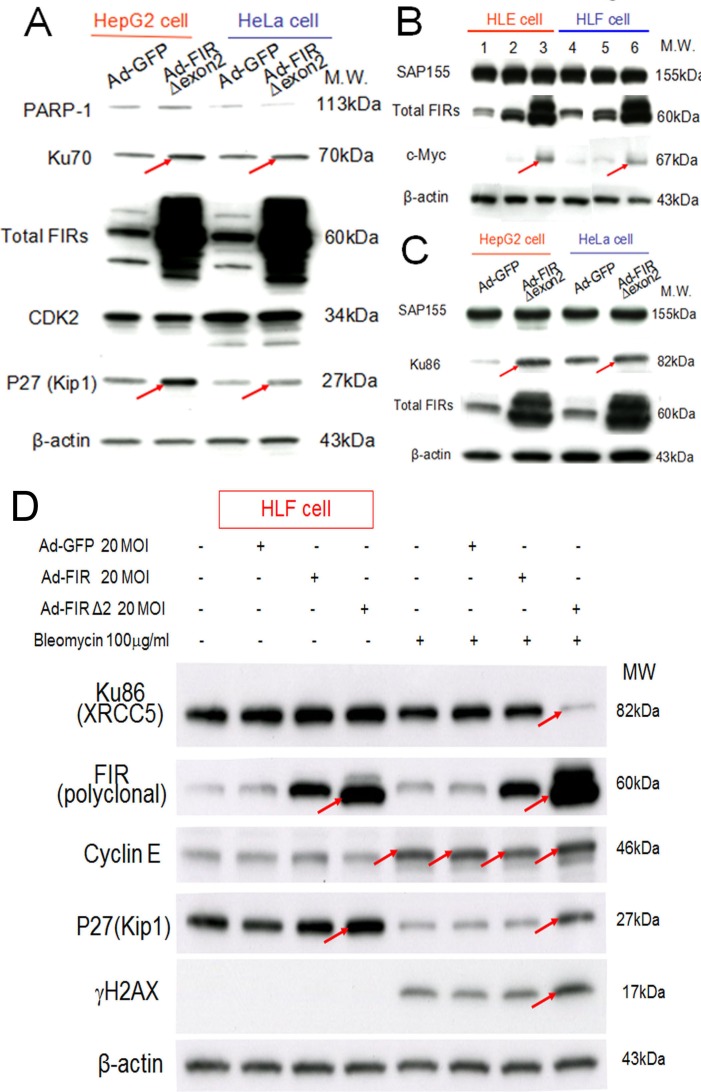
Ad-FIRΔexon2 increased Ku86/Ku70, P27Kip1, and c-Myc but not SAP155 in human HCC or HeLa cells Ad-FIRΔexon2 was transfected into HepG2, HLE, HeLa, and HLF cell lines. GFP adenovirus (Ad-GFP) was transfected as negative control. Whole-cell lysates were extracted 48 h after transfection and analyzed by Western blotting. (A) Ku70, P27Kip1, and related protein expression was examined in HepG2 and HeLa cell lines after transfection with 1.88 × 10^9^ VP/ml (50 MOI) of Ad-GFP or Ad-FIRΔexon2. (B) c-Myc and SAP155 expression was examined using HLE (lanes 1–3) and HLF (lanes 4–6) cell lines. Lanes 1 and 4 were transfected with 1.88 × 10^9^ VP/ml (50 MOI) of Ad-GFP, lanes 2 and 5 were transfected with 1.88 × 10^8^ VP/ml (5 MOI) of Ad-FIRΔexon2, and lanes 3 and 6 were transfected with 1.88 × 10^9^ VP/ml (50 MOI) of AD-FIRΔexon2. (C) Ku86 and SAP155 expression was examined in HepG2 and HeLa cell lines transfected with 1.88 × 10^9^ VP/ml (50 MOI) of the adenovirus vectors. (D) 100 μg/mL of BLM was treated with/without 20MOI (7.52 × 10^8^ VP/ml) of Ad-GFP, Ad-FIR or Ad-FIRΔexon2 transfection in HLF cells (hepatoblastoma cell line). Ku86/XRCC5, FIR (polyclonal antibody), cyclinE, P27/Kip1, γH2AX and β-actin (internal control) were examined.

siRNA against Ku86/Ku70 decreased FIR expression in HCT116 cells (Figure 3A, arrows). Additionally, total FIR expression was significantly suppressed by siRNA against Ku86 in HCT116, HepG2, and HLE cell lines (Figure 3B, arrows). Further, P27Kip1 was significantly suppressed by siRNA against Ku86 (Figure 3C, arrows). Moreover, both Ku86 and P27Kip1 were significantly suppressed by siRNA against FIR, and P27Kip1 was significantly suppressed by BLM-treatment alone (Figure 3D, arrows). Together, siRNA against Ku86 decreased FIR and P27Kip1, whereas siRNA against FIR decreased Ku86 and P27Kip1 protein expression. Note that P27* is an alternative splicing variant of authentic P27Kip1 [28], and P27* was also observed with siFIR (Figure 3D, arrowheads). These results indicated that FIR potentially interacts with Ku86, directly or indirectly, hence modifies P27Kip1 expression in cell-cycle control. BLM induced apoptosis in HeLa, HLE, and HLF cells at a concentration of 30μg/ml (Figures 4A, B). BLM treatment decreased Ku86/Ku70 and P27Kip1 proteins, whereas it increased γH2AX and cyclinE proteins (Figure 4C, Figure 5A, Figure 6D). Total FIR expression was suppressed by Ku86 siRNA in a dose-dependent manner (Figure 4D, arrows). These results indicated that sustained Ku86/Ku70 expression requires at least partly for FIR expression, accordingly FIR potentially has direct or indirect roles in DNA-damage repair. Furthermore, SAP155 expression was not significantly affected by siRNA against Ku86 (Figure 3A, Figure 4D). Thus, the effects on P27Kip1 and FIR expression were not directly related to Ku86 expression level, indicating that the mechanical or physical interaction of the SAP155/FIR/FIRΔexon2 complex may be essential for sustained expression of both Ku86 and P27Kip1.

### BLM suppressed SAP155 and increased FIR/FIRΔexon2 mRNA expression as well as the ratio of FIRΔexon2:FIR in HCC cells

Given that FIR engages in DNA-damage repair, BLM treatment should affect FIR expression at their mRNA or protein levels. This study showed that the expression levels of SAP155 and P27Kip1 decreased whereas that of γH2AX drastically increased after BLM treatment in dose-dependent manner (Figures 5A, arrows). More importantly, dose-dependent BLM treatment drastically increased FIR and FIRΔexon2 mRNA expression, as well as the ratio of FIRΔexon2:FIR mRNAs in HLE and HLF (Figures 5B). These results strongly suggest that BLM inhibited SAP155 or FIR function or mechanical interaction of FIR/SAP155 that is important for both P27Kip1 and FIR pre-mRNA splicing [11]. Since the extent of DNA damage is critical to cell fate, FIR, Ku86 and P27Kip1 should be coordinately regulated in response to the DNA damage pathway.

### FIRΔexon2 adenovirus vector (Ad-FIRΔexon2) increases Ku86/Ku70, *c-myc*, and P27Kip1 expression in HCC cells

If BLM inhibited SAP155 or FIR function or mechanical interaction of FIR/SAP155, that disturbs alternative splicing of FIR, FIRΔexon2 should have some effect to Ku86, Ku70 and P27Kip1 expression in DNA damage responses. Under this hypothesis, FIRΔexon2 adenovirus vector (Ad-FIRΔexon2) was prepared and treated to some cancer cells. Expectedly, Ad-FIRΔexon2 increased Ku70, P27Kip1, Ku86 in HepG2 and HeLa cells (Figure 6A, C, arrows), *c-myc* in HLE and HLF cells (Figure 6B, arrows). Thus, the alteration of the FIR:FIRΔexon2 ratio was influenced as DNA-damage repair response. Note Ad-FIRΔexon2 did not significantly alter the SAP155 expression (Figures 6B, C). Prominently, co-treated with BLM, Ad-FIRΔexon2 drastically suppressed Ku86/XRCC5 but increased P27Kip1 expression, and thus enhanced BLM-induced γH2AX expression (Figure 6D, arrows). These results also supported that altered P27Kip1 and Ku86 expression by FIRΔexon2 was related to mechanistic interaction among FIR/FIRΔexon2/SAP155 rather than simply an ectopic effect of FIRΔexon2 overexpression. Because FIR sustained Ku86 and vice versa, our results strongly suggest that alteration of the ratio FIR:FIRΔexon2 engages in DNA damage repair response.

### FIR/FIRΔexon2 engages in BLM-induced DNA damage repair by interfering with Ku86/Ku70 and P27Kip1 expression

**Figure 7:**
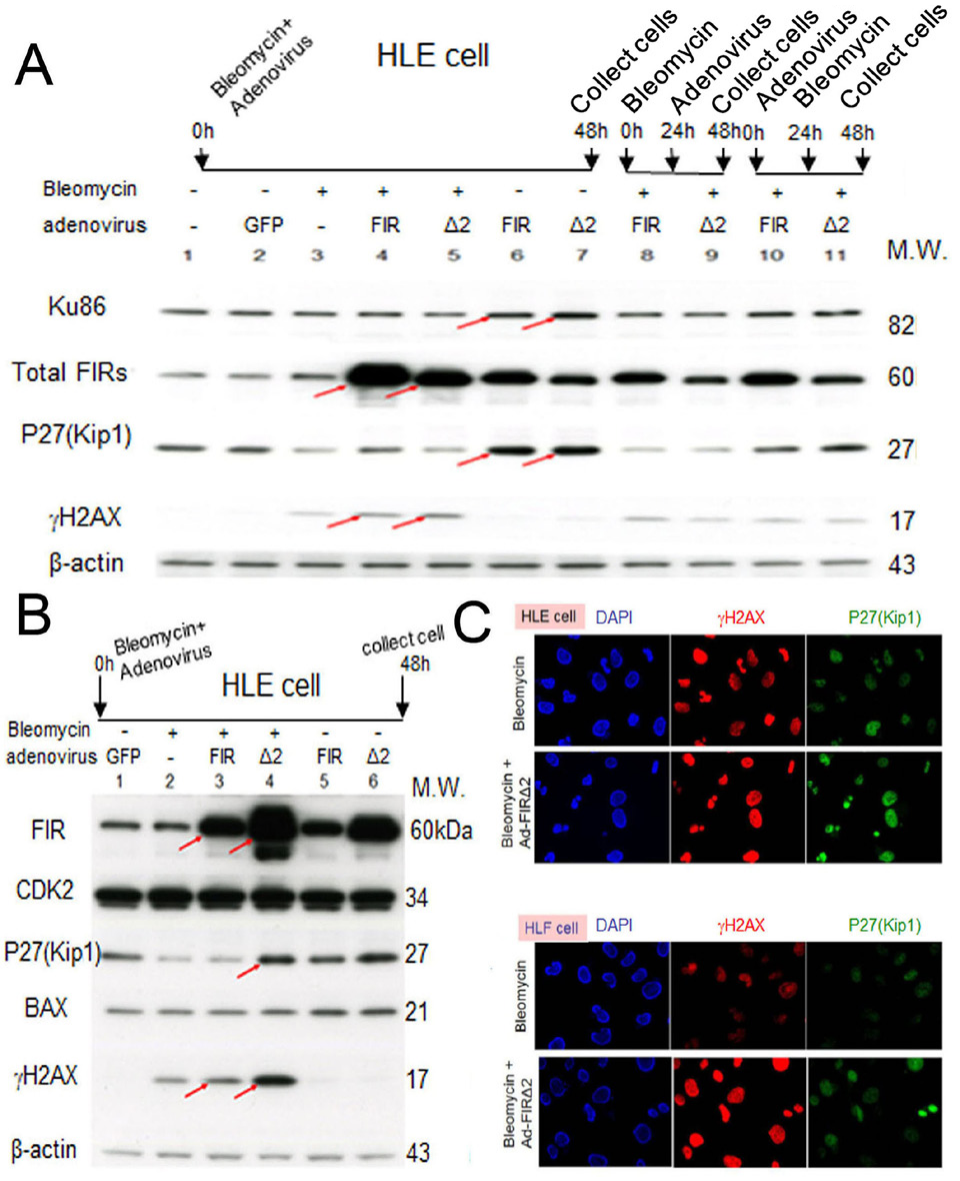
Ad-FIRΔexon2, rather than Ad-FIR, increased BLM-induced DNA damage as indicated by γH2AX in HLE and HLF cells (A) After 48h of bleomycin (BLM), Ad-FIR (FIR), Ad-FIRΔexon2 (Δ2) single treatment or co-treatment, whole-cell lysates of HLE cells were extracted and analyzed by Western blotting. For the co-treatment, BLM and Ad-FIR or Ad-FIRΔexon2 were co-treated at starting point and incubated for 48h (lanes 4 and 5). Ad-FIR (FIR) or Ad-FIRΔexon2 (Δ2) was added after 24h of BLM treatment, then incubated for another 24 h (lanes 8 and 9). After 24 h of Ad-FIR or Ad-FIRΔexon2 treatment, BLM was added, then incubated for another 24 h (lanes 10 and 11). BLM was added at a concentration of 30 μg/ml. Ad-GFP, Ad-FIR and Ad-FIRΔexon2 were added at a concentration of 3.76 × 10^8^ VP/ml (10 MOI). Lane 1, mock (untreated cell lysate); lane 2, Ad-GFP (GFP) as negative control; lane 3, BLM; lanes 4, 8 and 10, BLM and Ad-FIR; lanes 5, 9 and 11, BLM and Ad-FIRΔexon2. (B) HLE cells were treated with BLM (30 μg/ml) and/or Ad-GFP (3.76 × 10^8^ VP/ml; 10 MOI), Ad-FIR (3.76 × 10^8^ VP/ml; 10 MOI) or Ad-FIRΔexon2 (7.52 × 10^8^ VP/ml; 20 MOI) for 48 h, and whole-cell lysates were extracted and subjected to Western blotting. Lane 1, Ad-GFP (GFP); lane 2, BLM; lane 3, BLM with Ad-FIR; lane 4, BLM with Ad-FIRΔexon2; lane 5, Ad-FIR alone; lane 6, Ad-FIRΔexon2 alone. (C) P27Kip1 and γH2AX expression was examined in HLE and HLF cells by immunocytochemistry staining following BLM (100 μg/ml) treatment alone or with Ad-FIRΔexon2 (7.52 × 10^8^ VP/ml; 20 MOI).

Conversely, we examined whether Ad-FIRΔexon2 affects the ratio of FIRΔexon2:FIR, Ku86/Ku70, or P27Kip1 expression altered by BLM-induced DNA damage in HLE and HLF cells. P27Kip1 was significantly suppressed by BLM treatment (Figures 7A, B), and this suppression was rescued by Ad-FIRΔexon2 (Figure 7A, lanes 4, lanes 10 and 11; Figure 7B, lanes 3 and 4). BLM increased expression of γH2AX, a DNA-damage marker, and this expression was significantly increased further by Ad-FIRΔexon2 rather than by Ad-FIR (Figure 7A, B compare arrows). Ku86 and P27Kip1 were sustained their expression by Ad-FIR or Ad-FIRΔexon2 (Figure 7A, lanes 6 and 7). Together, these results indicate that FIR and FIRΔexon2 monitors or act as a molecular sensor of DNA-damage. Actually, Ad-FIR or Ad-FIRΔexon2 enhanced BLM-induced DNA damage by potentially interfering with Ku86/Ku70 (Figure 7A, compare lanes 4 and 5 to 3; Figure 7B, compare lanes 3 and 4 to 2, arrows). BLM-induced DNA damage affects the mechanical interaction of FIR/FIRΔexon2/SAP155 and monitors P27Kip1 expression for the evaluation of the extent of DNA damage (Figure 7C).

### BLM induces FIR in Mouse embryonic fibroblasts (MEFs) cells from wild-type control mice but not from FIR-hetero knockout mice

**Figure 8:**
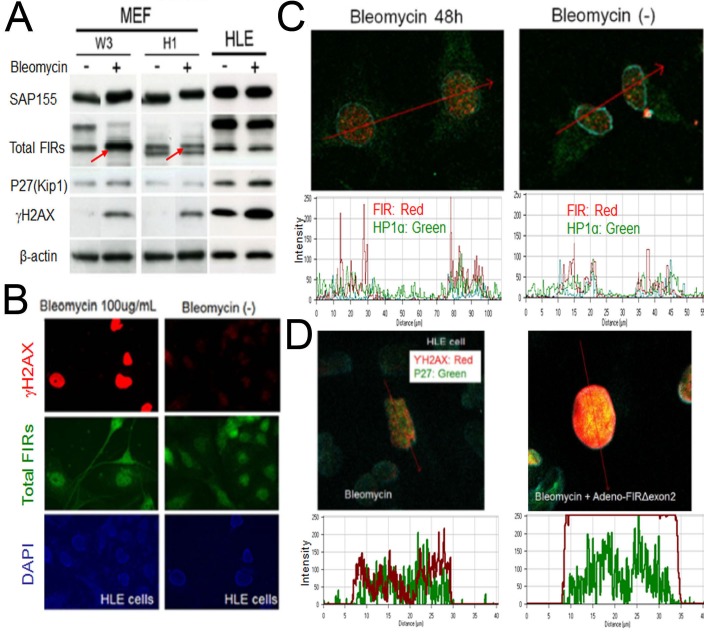
BLM induced DNA damage in MEFs (A) MEFs from wild-type C57BL/6 mice (W3) and from FIR-hetero knockout C57BL/6 mice (H1) were prepared (refer to materials and methods). MEF cells (W3 and H1 cells) were treated with or without 50μg/ml of BLM. HLE cells were also treated with same dosage of BLM as comparison. After 48 hours of incubation, whole cell lysates were extracted, then FIR, P27(kip1), SAP155 and γH2AX expressions were analyzed by western blotting. (B) HLE cells were treated with or without 100μg/ml of BLM. After 48 hours of incubation, cells were treated to immunocytochemistry for total FIRs and γH2AX expressions. (C) HeLa cells were treated with or without 100μg/ml of BLM. After 48 hours of incubation, FIR and ΗP1α expressions of cells were examined by immunocytochemistry. Expression levels were quantified by histogram. (D) HLE cells were treated with 100μg/ml of BLM alone or co-treated with 100μg/ml of BLM and Ad-FIRΔexon2 7.52×10^8^VP/ml (20 MOI). After 48 hours of incubation, immunocytochemistry was performed for γH2AX and P27 expressions. Expression levels were quantified by histogram.

If FIR is important for DNA-damage repair pathway, bleomycin-treatment should affect FIR expression. To examine this hypothesis, MEFs were prepared from FIR hetero-knockout mice (Supplementary Figure 1) and littermate control mice and treated with BLM. BLM increased FIR in the MEFs from wild-type C57BL/6 mice (W3) but not in those from FIR-hetero knockout C57BL/6 mice (H1) (Figure 8A, arrows). These results support that BLM activates transcription of FIR as well as its alternative splicing (Figure 5B). BLM-treatment (100 μg/mL for 48h) apparently increased γH2AX, a marker of DNA damage (Figure 8B) and increased the expression of FIR and HP1α (Figure 8C). Moreover Ad-FIRΔexon2 significantly enhanced BLM-induced DNA damage indicated by γH2AX and P27Kip1 expression (Figure 8D), coincide with the result stated above (Figure 6D).

### Ku86/Ku70, FIR, and PARP-1 protein expression is significantly decreased in cells with chromosomal instability (CIN) compared to those with microsatellite instability (MIN)

**Figure 9:**
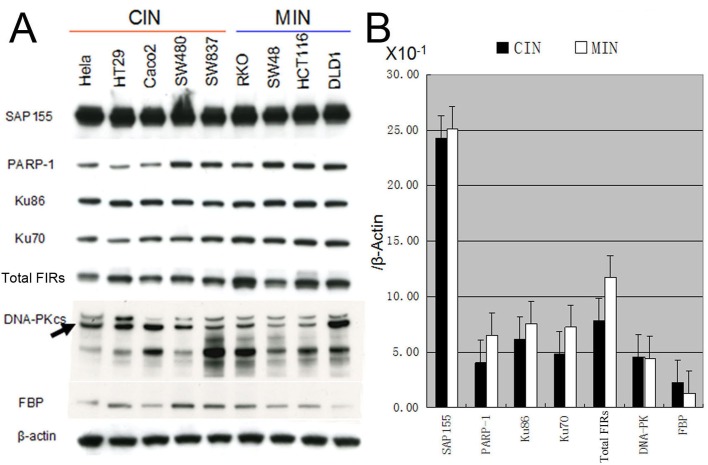
Expression of DNA-repair proteins in chromosomal instability (CIN) and microsatellite instability (MIN) cell lines (A) HeLa, HT29, CaCO2, SW480, SW837 cell lines were characterized as CIN cells and RKO, SW48, HCT116, DLD1 cell lines were characterized as MIN cells. (A) Expression of the DNA-repair proteins including PARP-1, Ku86/Ku70, DNA-PKcs, as well as proteins FIR, FBP and SAP155 was examined in those CIN and MIN cells by Western blotting. (B) Bands' intensities were quantified using TotalLab TL12 imaging analysis software (Shimadzu Co., Ltd. Kyoto, Japan) and the average band intensity of proteins normalized to the corresponding β-actin were shown. FBP indicated Far-upstream element binding protein.

**Figure 10:**
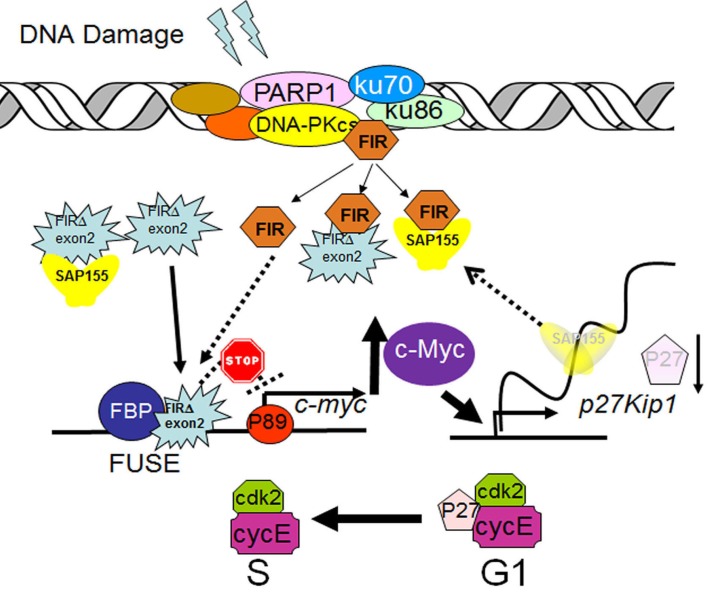
A model of alternatively splicing of FIR as a sensor for BLM-induced DNA-damage repair pathway by modulating c-Myc and P27Kip1 expression FIR potentially forms a complex with Ku86/Ku70 or DNA-PKcs. When DNA damage occurs, c-myc is transcriptionally regulated by FIR/FIRΔexon2, and P27Kip1 pre-mRNA splicing is modified through SAP155 expression. Thus, the mechanical interaction of FIR/FIRΔexon2/SAP155 potentially regulate c-Myc and P27Kip1 expression.

Eight human colorectal cancer cell lines and a human cervical cancer cell line (HeLa) were characterized for CIN, MIN status according to previous publications [29-34]. The expression of Ku86/Ku70, FIR, and PARP-1 was significantly decreased in CIN (HeLa, HT29, CaCO2, SW480, and SW837) cells compared with MIN (RKO, SW48, HCT116, and DLD1) cells (Figure 9). This result strongly supports the hypothesis that FIR expression is related to chromosomal instability due to its DNA-repair function. In summary, FIR/SAP155 complex accumulates at the break ends of BLM-induced DNA-damage sites, then alters *c-myc* and p27Kip1 expression (Figure 10).

### c-Myc is a key molecule that integrates the hypothetical network of nuclear proteins that expressed in both HLE and HLF cells

If mechanical or physical interaction between SAP155 and FIR is critical for cell survival against DNA damage, SAP155/FIR should interact with DNA-damage repair proteins. To examine the hypothetical network of nuclear protein pathways activated in DNA damage respose, nuclear proteins were extracted from HLE and HLF cells. The immunoprecipitates were analyzed and identified by in-gel tryptic digestion followed by liquid chromatography- tandem mass spectrometry (GeLC–MS)[35]. A total of 199 (53+146) and 419 (273+146) proteins were identified in HLE and HLF cell. 146 proteins were identified in both cells. 75 (19+56) and 148 (92+56) proteins were identified in HLE and HLF cell respectively, in the case of the restricting localization to nucleus. 56 proteins which were identified in both HLE and HLF cell nucleus (Supplementary Figure2A) were further analyzed for potential protein interaction network with transcription factors, including *c-myc*, HNF4-alpha, p53, and FKHR. Ku86/Ku70 forms a putative protein network controlled by *c-myc* (Supplementary Figure 2B). Proteins marked with red circles in Supplementary Figure 2B were identified in this study. These results indicated that DDX17, *c-myc*, and Ku70/86 were hypothetical SAP155/FIR-interacting proteins (Supplementary Table1).

## DISCUSSION

The present study indicated that the nuclear signaling of BLM-induced DNA-damage repair was transferred to the FIR/FIRΔexon2/SAP155 complex formation to regulate *c-myc* and p27Kip1 expression (Figure 10): (1) FIR/FIRΔexon2 potentially formed a complex with Ku86/Ku70, and both FIR/FIRΔexon2 and Ku86 were upregulated in human HCC tissues; (2) knockdown of FIR/FIRΔexon2 by siRNA decreased Ku86/Ku70 and vice versa; (3) BLM treatment significantly increased the expression of FIR and FIRΔexon2 mRNA, and Ad-FIRΔexon2 increased *c-myc*, Ku86, and P27Kip1 in HCC cells; (4) Ad-FIR/Ad-FIRΔexon2 delayed or increased BLM-induced DNA-damage repair by interfering with Ku86/Ku70 and, thus, increased P27Kip1 arrest of the cell cycle in HCC cells; and (5) FIR/FIRΔexon2/SAP155 formed a complex and disturbed *c-myc* regulation and P27 pre-mRNA splicing. In addition, BLM decreased P27Kip1; however, BLM and Ad-FIRΔexon2 co-treatment increased P27Kip1 protein expression. Co-treatment also increased γH2AX expression compared with BLM treatment alone, indicating that FIR/FIRΔexon2 adenovirus infection delayed BLM-induced DNA-damage repair by, at least in part, disturbing P27Kip1 pre-mRNA splicing (Figure 10).

The physical association of FIR, FIRΔexon2, and SAP155; the antagonized effects of FIR and FIRΔexon2 on *c-myc* transcription because FIRΔexon2 compete with FIR for FUSE binding site at *c-myc* promoter [11]. Further, the effects of SAP155/FIR/ FIRΔexon2 on the expression of P27, cdk2/cyclinE, and P89 provided novel information on a regulatory circuit that is important for cell growth and cancer progression [14]. Accordingly, FIR/FIRΔexon2/SAP155 complex formation potently disturbs alternative splicing of FIR pre-mRNA, and thus is a novel, *bona fide* molecular switch for *c-myc* gene expression [14]. In BLM-treatment, the increase of FIR/FIRΔexon2 mRNA and the ratio of FIRΔexon2:FIR was not correspond with that of total FIR protein in HLE and HLF cells (Figure 5). How should we explain this phenomenon? One possible reason is the time-lag of stability between FIR/FIRΔexon2 mRNA and total FIR protein. The other reason is due to the unveiled degradation pathway of total FIR protein. FIR potentially interacts with poly-ubiquitin ligase FBW7 (F-box and WD repeat domain-containing 7) (unpublished data). FBW7 has been characterized as an onco-suppressor protein in human cancers [36]. Further study is required to reveal the stability and degradation pathway of total FIR proteins. Ku86, the 86-kDa subunit of Ku encoded by human XRCC5, is a critical factor in the NHEJ DNA repair pathway [37]. Ku86 is overexpressed in HCC tissues and anti-Ku86/Ku70 autoantibodies in patient serum are a candidate biomarker for detecting early-stage hepatitis C virus-related HCC [27,38]. Previous studies also suggested that Ku86 variants may play a role in determining HCC susceptibility [39]. Ku86/Ku70 coimmunoprecipitated with PARP-1 [40] whereas PARP-1 and DNA-PKcs were identified by GeLC-MS as potent FIR-binding proteins (Supplementary Table 1). These results suggest that FIR has some direct or indirect roles in DNA-damage repair with Ku86. FIR/FIRΔexon2 adenovirus vectors increased expression of the DNA repair proteins Ku86/Ku70 and the cell-cycle regulator protein P27Kip1 (Figures 6 and 7).

Genetic instability include two major categories, one is microsatellite instability (MIN) which involves subtle changes in DNA sequences (faulty DNA repair), and the other is chromosomal instability (CIN) which is characterized by gains and losses of whole or parts of chromosomes and CIN is considered a driving force for tumorigenesis [30,41]. Single-stranded or double-stranded DNA breaks increase the susceptibility of chromosomal gross structural alterations which lead to CIN [30]. CIN is closely associated with intrinsic multidrug resistance of cancers [31]. In our study, FIR, Ku86/Ku70 and PARP-1 were significantly decreased in CIN cells compared with MIN cells (Figure 9). This result strongly supports the notion that FIR expression is closely related to chromosomal instability through engaging in DNA-repair mechanism.

SAP155 is associated with cyclinE and is efficiently phosphorylated *in vitro* by cyclinE–cdk2, a critical regulator of cell-cycle progression from G1 to S phases, suggesting a possible link between pre-mRNA splicing and cell-cycle progression in mammalian cells [42]. It has been demonstrated that SAP155 serves as a substrate for cyclinE–cdk2 *in vitro* and that its phosphorylation in the cyclinE complex can be inhibited by the cdk-specific inhibitor p21 [43,44]. Overexpression of p21 inhibits proliferation in mammalian cells and has been found to inhibit all cyclin–CDK complexes. Similar to p21, p27 inhibits cyclin–CDK complexes in G0/G1 [45].

This study indicated that the FIR/FIRΔexon2/SAP155 complex or mechanical interaction of these proteins is involved in DNA-damage repair pathway. The interactions of FIR/FIRΔexon2 and Ku86/Ku70 may provide new insight into the potential function of FIR/FIRΔexon2 in BLM-induced DNA-damage repair. This novel function of alternative splicing of FIR will contribute to further clinical studies for cancer treatment [46].

In summary, FIR/SAP155 complex accumulates at the break ends of BLM-induced DNA-damage sites, then alters *c-myc* and p27Kip1 expression (Figure 10). Altogether, our study indicated that the alternative splicing of FIR coordinates *c-myc*, P27Kip1/cyclinE and Ku86/XRCC5 expression and thus FIR is a novel *bona fide* molecular sensor for DNA damage.

## MATERIALS AND METHODS

### Human tissues and cancer cell lines

Human hepatocellular carcinoma (HCC) tissues from 15 patients were obtained at the occasions of tumor resection in the Department of General Surgery, Chiba University Hospital, Chiba, Japan. The clinical features of these 15 cases are summarized in Table 1. Written informed consent was obtained from each patient before surgery. All excised tissues were immediately placed in liquid nitrogen and stored at −80°C until analysis. Human hepatoblastoma cell lines (HLE, HLF, HepG2), human cervical cancer cell line HeLa, human colorectal cancer cell lines (HCT116, HT29, Caco2, SW480, SW837, RKO, SW48, DLD1) and human kidney embryonic cell line 293T cells were purchased from the American Type Culture Collection (ATCC) and stored in liquid nitrogen until use. Mouse embryonic fibroblasts (MEFs) cell lines were prepared from FIR hetero-knockout C57BL/6 mice (H1) and littermate control wild-type C57BL/6 mice (W3) (Supplementary Figure 1), then were cultured in Dulbecco's Modified Eagle Medium (DMEM) supplemented with 10% fetal calf serum (FBS; Invitrogen, Tokyo, Japan) and 1% penicillin–streptomycin. HLE and HLF cells were cultured in DMEM supplemented with 10% FBS. HepG2 cells were cultured in Iscove's Modified Dulbecco's Medium (IMDM) supplemented with 10% FBS. Remaining cell lines were cultured in IMDM supplemented with 10% FBS and 1% penicillin–streptomycin. Cells were grown at 37°C in a 5%-CO_2_ incubator.

**Table 1:** Clinical features of HCC patients examined in this study

No.	Age	Sex	Virus	Size (mm)	Adjacent Tissue	AJCC Stage
1	69	Male	HCV	25×22	LC	I
2	79	Female	not detected	22×15	LC	II
3	77	Female	HCV	18×16	LC	I
4	76	Female	HCV	14×8	LC	II
5	81	Male	not detected	48×45	CH	I
6	69	Male	not detected	70×70	Normal	III
7	65	Male	HCV	60×45	LC	III
8	76	Male	not detected	55×45	CH	III
9	80	Male	HCV	30×38	LC	II
10	58	Female	not detected	45×40	LC	II
11	61	Male	HCV	35×32	CH	II
12	65	Male	HCV	25×16	LC	II
13	75	Male	HCV	25×23	CH	I
14	75	Male	HCV	25×20	LC	II
15	79	Male	HCV	110×90	CH	III

Note: HCV, hepatitis C virus; LC, liver cirrhosis; CH, chronic hepatitis; AJCC, American Joint Committee on Cancer.

### Western blotting and antibodies

Culture medium was removed, and the cells were washed twice with cold (4°C) PBS, lysed with 1:20 β-mercaptoethanol and 2X sample buffer, and incubated at 100°C for 5 min. Whole-cell lysates were assayed for protein content (Bio-Rad, Hercules, CA, USA), and 10 μg of proteins were separated by SDS-PAGE on 7.5% or 10%–20% XV PANTERA gels and transferred onto polyvinylidene fluoride membranes using a tank transfer apparatus. The membranes were blocked with 0.5% skim milk in PBS overnight at 4°C. Antigens on the membranes were detected with enhanced chemiluminescence detection reagents (GE Healthcare UK Ltd., Buckinghamshire, UK). Membranes were incubated with primary antibodies for 1 h at room temperature, followed by three 10-min washes with 1XPBS/0.01% Tween 20. Membranes were then incubated with commercial secondary antibodies, followed by three 15-min washes with 1XPBS/0.01% Tween 20. The primary mouse monoclonal antibody against FIR C-terminus (Total FIRs 6B4) was prepared by Dr Nozaki [47] and preparation detail was described previously [11]. Details of other antibodies used in this study are listed in Supplementary Table 2.

### Stable transfection

Cells were transfected with plasmids using Lipofectamine 2000 reagents (Gibco BRL, MA, USA). For stable transfection, 5 × 10^4^ cells were transfected with FIR–FLAG or pcDNA3.1–FIRΔexon2 plasmids [11] and transferred to 10-cm dishes 48 h after transfection. The complete medium contained 400 μg/ml geneticin, 10% FBS, and 1% penicillin–streptomycin in IMDM. The complete medium was replaced every 4 days until geneticin-resistant colonies appeared. At least 30 clones were screened by immunoblotting and immunostaining with anti-FLAG and anti-FIR(6B4) antibodies to identify clones stably expressing FIR–FLAG or with anti-*c-myc* antibody to identify *c-myc* expression in cells stably expressing FIRΔexon2.

### Immunocytochemistry

Cancer cells were prepared for immunocytochemistry as described previously [9].

### FIR and FIRΔexon2 adenovirus vector preparation

Recombinant adenoviral vectors expressing full-length FIR proteins were constructed by homologous recombination in Escherichia coli using the AdEasy XL system (Stratagene). Preparation of the FIR and FIRΔexon2 adenovirus vectors were as previously described [11].

### Bleomycin (BLM) treatment

The DNA-damaging agent, bleomycin sulfate powder from Streptomyces verticillus, was purchased from Sigma–Aldrich (Tokyo, Japan; Lot no.BCBG6499V; PCode, 101203713), dissolved in distilled H_2_O at a concentration of 5 mg/ml, and stored at −20°C. HLE, HLF, HeLa, MEFs (H1 and W3) cells were seeded in 6-well plates at a density of 1–4 × 10^6^ cells/well in 2 ml of culture medium. The cells were incubated at 37°C/5% CO_2_ until confluence (approximately 24h). Immediately before drug treatment, the incubation medium was removed and replaced with fresh culture medium. Cells were treated with 1, 10, 30, 100 and 200μg/ml of BLM and/or 5 MOI (1.88 × 10^8^ VP/ml), 10 MOI (3.761 × 10^9^ VP/ml), 20 MOI (7.52 × 10^9^ VP/ml), 50 MOI (1.88 × 10^9^ VP/ml) of Ad-GFP, Ad-FIR, Ad-FIRΔexon2.

### Reverse transcription (RT)-PCR

Total RNA was extracted from cancer cells, organs and peripheral blood of sacrificed MEF wild-type or FIR hetero-knockout mice using QIAgene Miniprep Kit or PAXgene Blood RNA Kit (Qiagene, Tokyo, Japan). cDNA was synthesized from total RNA using a First Strand cDNA Synthesis Kit for RT-PCR (Roche, Mannheim, Germany). FIR cDNA was amplified using the cDNA as a template and the following forward and reverse primer pair: ATGGGCTTTGGAGATCCTCT and GTCCAATGTTGCTGGGTCTT, respectively. LightCyclerTM software version 3.3 (Roche) was used for real-time RT-PCR analysis. Reagents and PCR conditions were according to the manufacturer's instructions and as described previously [25].

### MTS assay (Cell proliferation assay)

Cells were cultured at a density of 5 × 10^4^ cells per well in flat-bottomed, 96-well plates. After a 24-h incubation at 37°C/5% CO_2_, cells were treated with BLM and/or FIR/FIRΔexon2 adenovirus vectors. The same volume of distilled H_2_O was used as a negative control. After a 48-h incubation at 37°C, CellTiter 96^®^ AQueous One Solution Reagent (Promega, Madison, WI, USA) was added to each well according to the manufacturer's instructions. Cell viability was determined by measuring the absorbance at 490 nm using a 550 Bio-Rad plate reader.

### Apoptosis assay

HLE, HLF, and HeLa cells were cultured at a density of 2 to 5 × 10^4^ cells per well in flat-bottomed, 96-well plates for approximately 24 h before drug treatment. BLM or Ad-FIR/Ad-FIRΔexon2 was added to each well separately. After 48 h of culture, cell viability was determined using an APOPercentage Apoptosis Assay™ Kit (Funakoshi Co., Ltd., Tokyo, Japan) according to the manufacturer's instructions. Briefly, 100 μl of 0.4% gelatin was added to each well and allowed to settle for at least 10 min. Wells were seeded with 2–5 × 10^4^ cells in 200 μl of culture medium. The cells were incubated at 37°C/5% CO_2_ until confluence (approximately 24 h). The incubation medium and gelatin were removed, the cells were rinsed with fresh medium, 100 μl of fresh culture medium containing 5 μl of APOPercentage Dye and the apoptotic inducer/inhibitor was added to the center of each well, and the incubation was continued for 30 min. The culture medium/dye mixture was removed with a syringe, and the cells were gently washed twice with 200 μl of PBS/well. The cells were immediately observed under an inverted microscope, and at least three photomicrographs of a representative area of each well were obtained.

### siRNAs against FIR, SAP155, Ku86, and Ku70

FIR, SAP155, Ku86, and Ku70 siRNA duplexes were purchased from Sigma–Aldrich. Transient transfection of siRNAs was performed using Lipofectamine 2000 (Invitrogen) according to the manufacturer's instructions. The transfected cells were cultured for 48 h at 37°C in a CO_2_ incubator. The target sequences for the siRNAs are listed in Supplementary Table 3.

### Pull-down assays with anti-Ku86 antibody-conjugated beads and protein pathway analysis

Nuclear proteins extracted from HLE or HLF cells were immunoprecipitated with anti-Ku86 antibody-conjugated Dynabeads™ (Life Technologies, Carlsbad, CA, USA) according to the manufacturer's instructions [11]. Briefly, cells were homogenized using a Dounce homogenizer in 10 volumes of cold buffer [20 mM HEPES (pH 7.4), 250 mM sucrose, 0.05% NP-40, and protease inhibitor cocktail (Roche Diagnostics)]. The homogenate was centrifuged at 600×g at 4°C for 10 min. After the pellet was washed twice with the same cold buffer, it was solubilized in 10 volumes of RIPA buffer (50 mM Tris, 150 mM NaCl, 0.1% SDS, 0.5% deoxycholic acid sodium salt, 1% NP-40, and protease inhibitor cocktail) and sonicated twice for 10 s. The nuclear fraction (400 μl) was immunoreacted with anti-Ku86 antibody and anti-mouse IgG antibody (Supplementary Table 2) conjugated to Dynabeads (100 μl) at 4°C overnight. The Dynabeads were then washed five times with RIPA buffer (500 μl) and eluted with 100 mM glycine (40 μl; pH 2.0; WAKO Pure Chemical Industries Ltd., Osaka, Japan) at 4°C for 10 min. The immunoprecipitates were analyzed by GeLC-MS [29]. Fifty-six proteins were identified as potential Ku86-binding nuclear proteins in both HLE and HLF cells (NCBI annotation) (Supplementary Figure 2A). The highest probability protein network was analyzed with the protein pathway software Metacore™ by adding transcription factors such as *c-myc*, HNF4-alpha, p53, and FKHR (Supplementary Figure 2B).

### MEFs of FIR hetero-knockout mice

FIR hetero-knockout [FBP interacting repressor (Puf60) floxed] mice were established, registered, and made available at the National Institute of Biomedical Innovation (http://animal.nibio.go.jp/j_fir.html) and the experimental animal division of the RIKEN Bioresource Center, Japan (RBRC No. RBRC05542; http://www2.brc.riken.jp/lab/animal/search.php). Briefly, two loxP sites were inserted upstream of FIR exon 3 and downstream of exon 5, and a PGK-neo cassette and loxP site were inserted downstream of exon 5. Conditional FIR-deficient mice could be generated by crossing with tissue-specific Cre mice to give Mus musculus C57BL/6-Puf60<tm1>/CU(Supplementary Figure 1).

### Statistical analysis

Statistical significance of the differences in numerical data were assessed by the the Student's t-test and the Wilcoxon test.

## SUPPLEMENTARY FIGURES AND TABLES




