# Supplementary material for: Alternative splicing of FBP-interacting repressor coordinates c-Myc, P27Kip1/cyclinE and Ku86/XRCC5 expression as a molecular sensor for bleomycin-induced DNA damage pathway
Source: Oncotarget. 2013 Dec 21;5(9):2404–17. doi: 10.18632/oncotarget.1650 (PMC4058014; doi:10.18632/oncotarget.1650)
Supplement: Supplementary file 1 [file oncotarget-05-2404-s001.pdf]

Alternative splicing of FBP-interacting repressor coordinates c-Myc, P27Kip1/cyclinE and Ku86/XRCC5 expression as a molecular sensor for bleomycin-induced DNA damage pathway – Rahmutulla et al

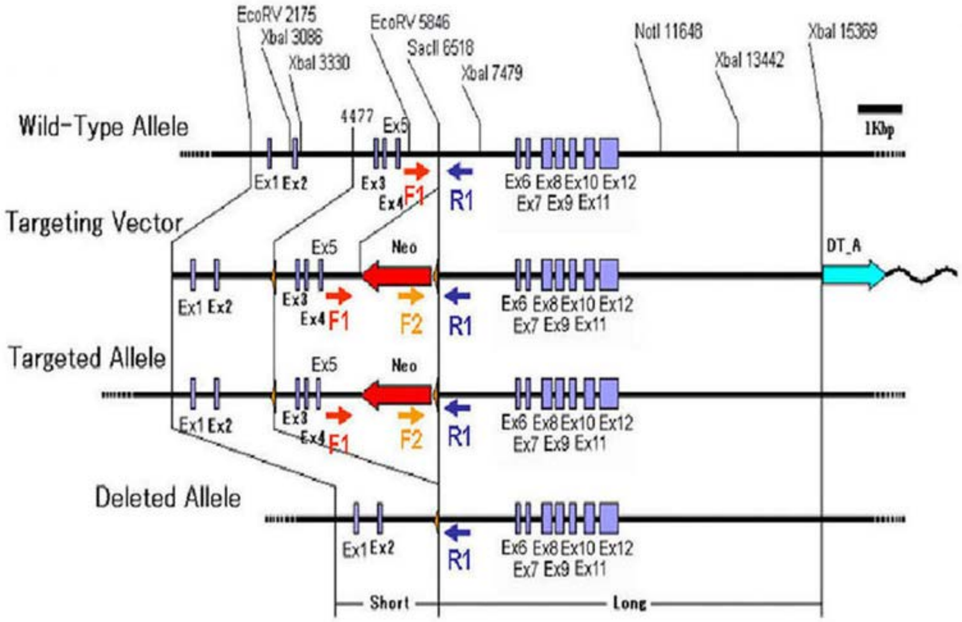

Supplementary Figure 1: Mouse embryonic fibroblasts (MEFs) prepared from FIR hetero-knockout mice. A schematic view of the construction of FIR hetero-knockout mice shows that two loxP sites was inserted between upstream of FIR exon 3 (Ex3) and downstream of exon5 (Ex5), and a PGK-neo cassette and loxP site were inserted downstream of exon 5 (Ex5). MEFs were prepared from both the FIR hetero-knockout mice and the littermate control mice.

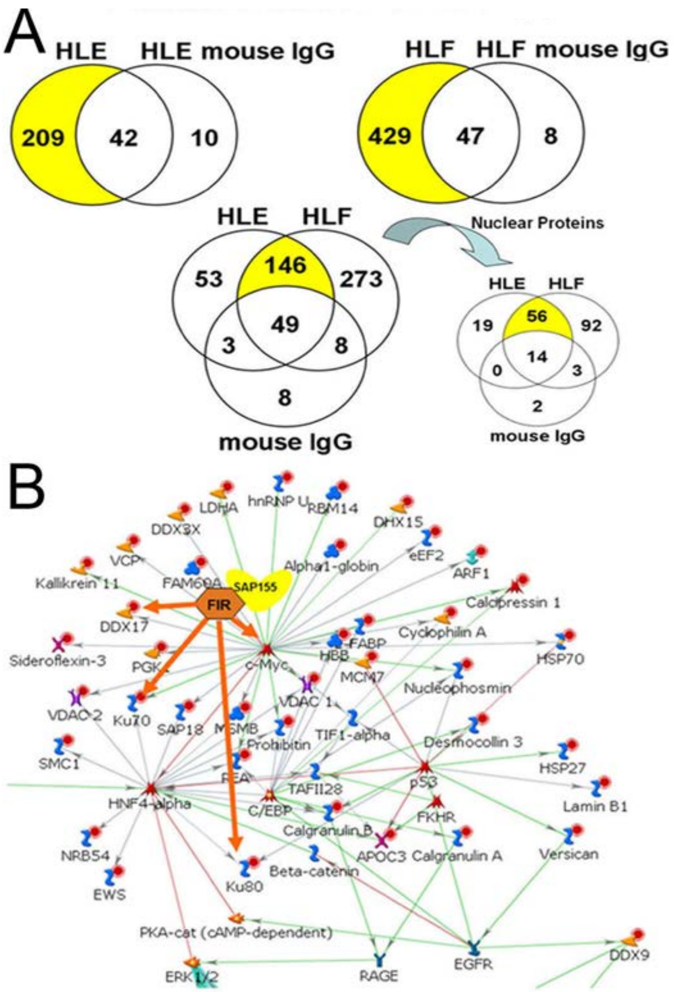

Supplementary Figure 2: Ku86(Ku80)/Ku70 is involved in a hypothetical protein pathway network controlled by c-Myc. Nuclear proteins were immunoprecipitated from HLE or HLF nuclear fractions with anti-Ku86 antibody or mouse IgG-conjugated Dynabeads™. The immunoprecipitates were analyzed and identified by GeLC-MS. Interacting proteins with Ku86 were identified 199 (53+146) and 419 (273+146) proteins in HLE and HLF cell, respectively. 146 proteins were identified in both cells. 75 (19+56) and 148 (92+56) proteins were identified in HLE and HLF cell respectively, in the case of the restricting localization to nucleus (NCBI annotation). (B) The 56 proteins were analyzed with protein interaction network. The transcriptional factors of potential interaction with 56 proteins were c-Myc, HNF4-alpha, p53, and FKHR. Proteins marked with red circles were identified in this study. DDX17, c-Myc, and Ku70/80 were potent SAP155/FIR-interacting proteins.
